# The Effectiveness of Life Skills-Based School Violence Prevention Programs in Secondary Education: A Systematic Review and Meta-Analysis

**DOI:** 10.3390/children13091128

**Published:** 2026-08-23

**Authors:** Konstantina Trimmi, Theodoros Fouskas, Vasiliki Yotsidi, Tonia Vassilakou, Aikaterini Papatheochari, Vana Gkora, Kyriakoula Merakou

**Affiliations:** 1 Department of Public Health Policy, School of Public Health, University of West Attica, 11521 Athens, Greece; tfouskas@uniwa.gr (T.F.); tvasilakou@uniwa.gr (T.V.); kmerakou@uniwa.gr (K.M.); 2 Department of Psychology, Panteion University of Social and Political Sciences, 17671 Athens, Greece; 3 Unified Special Vocational Junior High School and High School of Chalkida, 34150 Chalkida, Greece; 4 Net Media Lab & Mind & Brain R&D, Institute of Informatics & Telecommunications, National Centre of Scientific Research “Demokritos”, 15341 Agia Paraskevi, Greece

**Keywords:** school violence, life skills, adolescence, prevention, secondary education

## Abstract

**Highlights:**

**What are the main findings?**

• Life skills-based school violence prevention programs showed an overall small-to-moderate statistically significant reduction in violent behaviors among secondary school students, but effects exhibited a very high degree of variation across studies.
• Explanatory moderator analyses (outcome type, study design, and risk of bias), did not account for the observed heterogeneity, indicating that no specific study or intervention type was consistently more effective than others.

**What are the implications of the main findings?**

• While life skills education holds potential for reducing school violence, the high variability in outcomes means program success cannot be guaranteed for any single implementation.
• Future research requires pre-registered, high-quality randomized trials with standardized outcome measurement and longer follow-up periods to identify active intervention components.

**Abstract:**

**Background**: School violence is a major public health concern, with bullying alone affecting up to 50% of adolescents worldwide. Life skills-based programs have been proposed as a promising preventive approach, yet evidence for their effectiveness in secondary education remains limited. This systematic review and meta-analysis aims to synthesize the existing evidence on the effectiveness of life skills-based violence prevention programs among adolescents aged 12–18. **Methods**: A systematic literature search was conducted across five databases (PubMed, Scopus, ERIC, CINAHL, and PsycInfo) in accordance with PRISMA 2020 guidelines (INPLASY202650168). Interventional studies published between 2005 and 2025 that targeted secondary school students were eligible. Risk of bias was assessed using the Cochrane RoB2 (for RCTs/cRCTs) and ROBINS-I tools. A three-level random-effects meta-analytic model with Robust Variance Estimation was employed to pool Hedges’ g effect sizes. **Results**: Nineteen studies met the eligibility criteria, with 18 contributing 56 effect sizes to the meta-analysis. The pooled effect size was statistically significant (*g* = −0.2652, 95% CI [−0.4879, −0.0425], *p* = 0.0196), indicating an overall small reduction in violent behaviors. However, a high degree of variation in effects was observed across studies (Q(55) = 2263.58, *p* < 0.001; I^2^ = 99.23%). None of the examined moderators—including outcome type, study design, and risk-of-bias tier—significantly explained this heterogeneity. The sensitivity analysis, excluding one highly influential study, yielded a smaller pooled effect (g = −0.1422, 95% CI [−0.2246, −0.00598]) while maintaining high heterogeneity (I^2^ = 92.82%). **Conclusions**: Although life skills-based programs demonstrate a statistically significant overall average effect in reducing school violence among adolescents, this finding must be interpreted with extreme caution given the high degree of variation in effect sizes across contexts and the fact that prediction intervals span across zero. Because moderator analyses revealed no significant differences across intervention types, study designs, or outcome categories, no specific program type can be identified as consistently more effective. Future primary research must prioritize prospectively registered trials with multi-informant assessments to establish true intervention effectiveness.

## 1. Introduction

Violence in school-based settings constitutes one of the most pressing public health concerns of our time. Interpersonal violence is among the top five leading causes of mortality for adolescents worldwide [1]. Broadly defined, school violence refers to any act of physical, relational, or psychological aggression perpetrated within—or in association with—school contexts, with the intent to cause harm to another individual. Several types of violence can be identified: Physical aggression includes disruptive contact behaviors such as pushing, punching, and object throwing with the intent to cause pain [2]. On the other hand, relational aggression aims to emotionally hurt or devalue an individual through the use of hostile language [3]. Bullying is one the most researched forms of violence in a school setting. It is described as the systematic abuse of an individual (whether physical or relational) where there is a notable imbalance of power between the aggressor and the victim [4]. Prevalence estimates suggest bullying affects up to 50% of students [5,6], with substantial effects both for the aggressor and the victim, including emotional distress, avoidance, heightened risk of suicide, and criminality in adulthood [7].

Schools constitute ideal environments for violence prevention as they represent microsocieties in which most children and adolescents participate. Additionally, research indicates that aggression peaks in middle school years (ages 11–14) [8,9], highlighting the importance of preventative measures during this developmental period.

According to the World Health Organization, life skills are defined as “abilities for adaptive and positive behavior that enable individuals to deal effectively with the demands and challenges of everyday life” [10] and can be broadly categorized as: decision-making skills, interpersonal and communication skills, and self-management skills [11]. As anti-social behavior, lack of self-control, and poor academic skills have been identified as risk factors for youth violence perpetration [12], life skills enhancement through the educational system is considered a crucial and effective preventive measure.

A variety of life skills targeting programs have been successfully implemented [13], resulting in reduced overall violence. The magnitude and cost-effectiveness of an intervention appear to be higher the earlier it is implemented [13]. As such, most of the literature focuses on programs aimed at preschool or elementary school level, leaving significant gaps in knowledge regarding the effectiveness of preventive programs in middle and high school settings. This review aims to address this issue by providing a comprehensive collection of reported effects during this period—across a broad range of intervention designs and outcome variables—and attempts to provide an overall estimate of the effectiveness of school-based violence prevention programs on adolescents.

## 2. Materials and Methods

### 2.1. Search Strategy

A thorough literature search was performed on the 16 June 2025 across 5 different databases (PubMed, Scopus, ERIC, CINAHL, and PsycInfo), in accordance with the PRISMA 2020 guidelines [14], and was registered with Inplasy (registration number: INPLASY202650168). The PICOS algorithm that was applied is shown in Table 1. The search string was adapted for each of the 5 databases using a combination of a free text search and keywords retrieved from each database’s thesaurus. The free text search included the terms “school-based”, “prevention”, “program”, “intervention”, “practice”, “module”, “workshop”, “strategy”, “approach”, “violence”, “violent”, “aggression”, “stress”, “life skills”, “empathy”, “self-awareness”, “compassion”, “sympathy”, “critical thinking”, “creative thinking”, “decision making”, “problem solving”, “emotion management”, “emotional control”, “emotional intelligence”, “social emotional learning”, “relationship management”, “relationship building”, “stress management”, “stress regulation”, “resistant to social pressure”, “communication skills”, “interpersonal skills”, “social competence”, “emotional competence”, “emotion regulation”, “self-regulation”, “coping skill”, “resilience”, “prosocial behaviour”, “behaviour regulation”, “conflict resolution”, “anger management”, “secondary education”, “adolescent”, “teenager”, “adolescence”, “teen”, “puberty”, “student”, “high school”, “effectiveness”, “impact”, and “outcome” and was consistent across all databases. Full search strings can be found in the Supplementary Materials. Only peer-reviewed studies published in English or Greek between 2005 and 2025 were retrieved for the purposes of this review.

#### 2.1.1. Eligibility Criteria

The present review focuses on interventional studies in adolescents, assessing the effectiveness of intervention programs on violent behaviors. All collected studies were imported in a reference manager (Zotero), where duplicates were automatically removed. Screening was performed separately by 2 researchers in a two-step process: title and abstract screening, followed by full text search. Studies meeting the inclusion criteria shown in Table 2 were included in this review, while those meeting the exclusion criteria shown in Table 3 were excluded.

#### 2.1.2. Quality Assessment

Randomized trials were assessed using the Cochrane revised RoB2 tool [15], which evaluates studies based on 5 domains: randomization, deviation from intended intervention, missing data, outcome measurements, and selective reporting of results. Specifically, for cluster-randomized controlled trials (cRCTs), the revised Cochrane RoB 2 tool explicitly adapted for cluster-randomized trials was applied, incorporating considerations for timing of identification/recruitment of participants and cluster-level randomization dynamics. Nonrandomized interventions were assessed using the Cochrane revised ROBINS-I V2 tool, which evaluates studies based on 6 domains: confounding, classification of intervention, selection into the study, missing data, outcome measurements, and selective reporting.

#### 2.1.3. Data Collection

Descriptive data was extracted from each study in a spreadsheet format, including author name, publication year, country, study design, sample size and age, outcome variables, and statistical outcomes. Study pre-registration status was also recorded (e.g., trial registry IDs from ClinicalTrials.gov or ISRCTN). When no registration was explicitly reported in the manuscript or public registries, the study was classified as not pre-registered.

Standardized mean differences were calculated from reported group means and standard deviations at post-intervention or follow-up. When studies reported alternative statistics (e.g., odds ratios, regression coefficients, or standardized effects), these were converted to standardized mean differences using established formulas to ensure comparability across studies [16,17]. All effect sizes were expressed as Hedges’ g to correct for small-sample bias. When confidence intervals were reported instead of standard errors, standard errors were derived from the width of the confidence interval. Several studies contributed more than one effect size, corresponding to multiple outcome measures (e.g., physical aggression, bullying perpetration/victimization, etc.) reported within the same sample, producing statistical dependence among effect sizes nested within the same study.

#### 2.1.4. Meta-Analysis

To account for the dependency of multiple effect sizes nested within the same study, a three-level random-effects meta-analytic model was employed using the metafor package [18] in R. Robust Variance Estimation (RVE) was applied on top of the three-level model using the clubSandwich package [19]. Heterogeneity was assessed using Cochran’s Q-test and by decomposing total variance into the proportion attributable to between-study and within-study levels. Sensitivity analyses were performed by a leave-one-out procedure in which each study was sequentially removed and the model re-estimated. Prediction intervals (PIs) of 95% were estimated for a new comparable study for both the overall and sensitivity models (see results) using the predict() function in the metafor package. Publication bias was assessed on an aggregated (study-level) model using three complementary methods: trim-and-fill, Egger’s regression test, and Begg’s rank correlation test [20].

## 3. Results

### 3.1. Description of Eligible Studies

**Figure 1:**
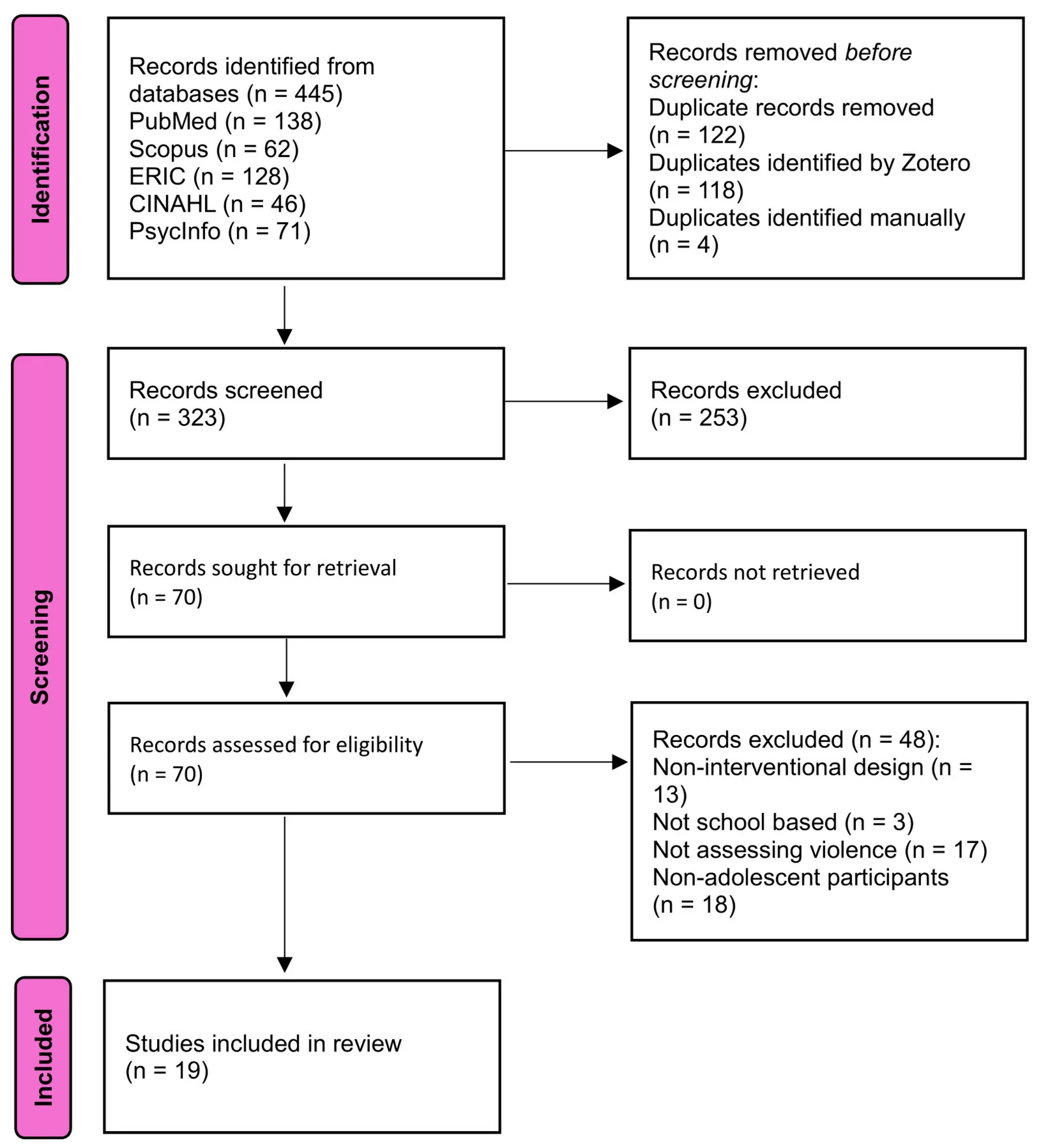
Prisma flow diagram for study selection.

A total of 445 studies were retrieved from all databases (PubMed: 138, Scopus: 62, ERIC: 128, CINAHL: 46, PsycInfo: 71). Automatic deduplication from Zotero removed 118 studies, while four duplicates were removed manually. From the remaining 323 studies, 253 were excluded during title and abstract screening and 51 during full text search due to meeting some of the exclusion criteria, leading to 19 eligible studies for this review. A complete PRISMA flowchart is shown in Figure 1.

More than half of the eligible studies (10/19, 52.6%) were conducted in the US. Only two studies came from Europe, more specifically from Spain, while the remaining seven studies were from Brazil, Canada, India, Japan, Korea, Pakistan, and Thailand (one study each). Across the desired period, the publications were relatively evenly distributed, with a peak of four during 2020 and no studies published during 2010–2011 or 2021–2022.

All of the studies had an interventional focus, with most (15/19) providing dedicated control group comparisons either as quasi-experimental or randomized controlled trial design. The remaining studies followed a pretest–post-test protocol, either as repeated measures within the same group, or more complicated designs like the “extended age cohort”, where same-age students are compared across periods of time [21]. Sample size varied greatly across studies, from small experimental interventions with 45 students to large-scale program implementations with over 100,000 participants. The age range of students differed from study to study with more emphasis given to young adolescents (12–13 years old). A summary of eligible studies characteristics is shown in Table 4. Regarding trial pre-registration, only 3 out of the 19 included studies (15.8%) reported prospective trial pre-registration: Wolfe et al. [22] (ISRCTN76259226), Niolon et al. [23] (NCT01672541), and Niolon et al. [24] (NCT01672541). The remaining 16 studies (84.2%) did not report pre-registration.

Quality assessment of the studies showed medium to high risk of bias (Table 5 and Table 6), which is often observed across school-based interventions. The key factors driving this result were: (i) the self-reporting assessment of the outcome, which was present in all studies, most often without blinding the student to the treatment condition; (ii) high attrition rates, especially in longitudinal studies; and (iii) uncontrolled confounding in quasi-experimental studies.

Most of the included studies reported at least partial support for the effectiveness of life skills-based programs in reducing school violence outcomes (Table 7). Interventions targeting dating violence and bullying-related outcomes reported the most consistently positive results while studies assessing general aggression produced mixed results. Only three studies included post-intervention follow-up assessments beyond the immediate post-test (e.g., Niolon et al., 2024 [22,24,25]). Given the scarcity of follow-up reporting across the literature, long-term intervention persistence could not be quantitatively meta-analyzed or formally synthesized.

The life skills components most consistently associated with positive outcomes across studies were emotional regulation, conflict resolution and problem solving, communication, and empathy. Prosocial skill development, self-control, and confidence were also prominent in effective programs. Three studies specifically showcased the mediation effect of emotional regulation [26], decision-making skills [27], and cooperative peer relationships [28] on the success of program implementations.

Gender moderation is a recurring observation across studies. Wolfe et al. [22] and Sullivan et al. [29] report stronger intervention effects for boys, while Karmaliani et al. [30] and Sharma et al. [25] report greater intervention effects for girls. It should be noted that there are also studies that tested for gender moderation with no conclusive results [31,32]. Age also emerged as a moderating variable in some studies, with older adolescents demonstrating greater gains [25], while Limber et al. [21] found grade-dependent differential effects within the same program. These patterns require further investigation, possibly hinting that intervention design and implementation should be sensitive to developmental stage and gender.

**Table 4:** Eligible study characteristics (CG = control group, IG = intervention group).

Authors	Year	Country	Study Design	Sample Size	Age of Participants	Targeted Life Skills
Ando [33]	2007	Japan	Quasi-experimental; nonequivalent control group; delayed intervention	104; First treatment = 52, Delayed treatment = 52	12–13 (7th grade)	Problem solving, self-management and self-control skills, communication, peer resistance, and conflict resolution.
Wolfe [22]	2009	Canada	Cluster RCT	1722 (CG n = 754, IG n = 968)	14–15	Relationship skills to promote safer decision making with peers and dating partners; negotiation, delay, and refusal skills; exercises to define and rehearse responsibilities associated with healthy relationships; and interpersonal and problem-solving skills.
Castillo-Gualda [26]	2017	Spain	Quasi-experimental	476 (CG n = 146, IG n = 330)	11–15 (12.8)	Emotional perception, emotional expression, emotional facilitation, emotional understanding, and emotional regulation.
Niolon [23]	2019	USA	Cluster RCT	2349 (CG n = 1192, IG = 1157)	12 (6th-8th grade)	Developing respectful and healthy relationships and assisting youth in practicing healthy relationship skills.
Duncan [34]	2017	USA	Cluster RCT	1130	3rd-8th grade	Feeling good about oneself; self-control and social skills.
Van Ryzin & Roseth [28]	2018	USA	Cluster RCT	1460	7th grade	Improved peer relations and cooperative learning.
Valente [27]	2020	Brazil	Cluster RCT	6691 (CG n = 3243, IG n = 3148)	12–13 (7th–8th grade)	Decision-making skills, social and interpersonal skills, and personal skills.
Taylor [32]	2015	USA	Cluster RCT	2655	6th–7th grade	Awareness, understanding, and setting boundaries
Frank [35]	2014	USA	Quasi-experimental; nonequivalent control group; pretest–post-test	49	9th–12th grade	Social-emotional health and physical wellness, stress management, body and emotional awareness, self-regulation, and building healthy relationships.
Limber [21]	2018	USA	Quasi-experimental; extended age cohort	n1 = 70,998 n2 = 31,675	3rd–11th grade	Empathy.
Sulivan [29]	2016	USA	Cluster RCT	231 (CG n = 117, IG n = 114)	11–15 (12.6)	Empathy and emotion management (social and emotion regulation).
Niolon [24]	2024	USA	Cluster RCT	2840 (CG n = 1425, IG n = 1415)	9th–11th grade	Healthy relationship skills or positive relationship skills.
Benítez-Sillero [31]	2020	Spain	Quasi-experimental	764	12–19 (14.8)	Collaborative work, self-esteem, empathy, self-control, resilience, and discrimination.
Thompkins [36]	2014	USA	Quasi-experimental	1112	9th–10th grade	Conflict resolution skills and prosocial skills.
Sharma [25]	2020	India	Quasi-experimental	203 (CG n = 108, IG n = 98)	8th grade	Emotional intelligence, social skills, and empathy.
Karmaliani [30]	2020	Pakistan	Cluster RCT	1752	12–14 (6th–8th grade)	Confidence, communication, empathy, coping with negative emotions, resilience, cooperation, leadership, critical thinking, and conflict resolution.
Massey [37]	2008	USA	Quasi-experimental	446 (CG = 299, IG = 147)	Secondary school	Self-control, social competency, positive peer relationships, and interpersonal problem solving skills.
Konguswan [38]	2012	Thailand	Quasi-experimental; nonequivalent control group; pretest–post-test	45	12–15 (13.33)	Self-awareness, self-investigation, self-examination, active listening, interpersonal relationship skills, coping with emotions and stress, problem solving skills, and social responsibility skills.
Suh [39]	2023	Korea	Quasi-experimental	73 (CG n = 35, IG n = 38)	15–16 (10th grade)	Self-esteem, assertiveness, emotional expression, empathy, cooperation, and active listening skills.

**Table 5:** Quality assessment of randomized controlled trials using Cochrane RoB2.

Study	D1: Randomization	D2: Deviations	D3: Missing Data	D4: Measurement	D5: Reporting	Overall
Wolfe et al. [22]	Some concerns	Low	Some concerns	Some concerns	Low	Some concerns
Niolon et al. [23]	Some concerns	Low	Some concerns	Some concerns	Low	Some concerns
Duncan et al. [34]	Some concerns	Low	Some concerns	High	Some concerns	High
Van Ryzin & Roseth [28]	Some concerns	Low	Low	Some concerns	Low	Some concerns
Valente et al. [27]	Low	Low	Some concerns	Some concerns	Some concerns	Some concerns
Taylor et al. [32]	Some concerns	Some concerns	Low	Some concerns	Low	Some concerns
Sullivan et al. [29]	High	Some concerns	Some concerns	Some concerns	Some concerns	High
Niolon et al. [24]	Some concerns	Low	Some concerns	Some concerns	Low	Some concerns
Karmaliani et al. [30]	Low	Low	Low	Some concerns	Low	Low

**Table 6:** Quality assessment of quasi-experimental studies using Cochrane ROBINS-I V2.

Study	D1: Confounding	D2: Classification	D3: Selection	D4: Missing Data	D5: Measurement	D6: Reporting	Overall
Ando et al. [33]	Serious	Low	Moderate	Moderate	Serious	Moderate	Serious
Castillo-Gualda et al. [26]	Serious	Low	Moderate	Moderate	Serious	Moderate	Serious
Frank et al. [35]	Critical	Low	Serious	Moderate	Serious	Moderate	Critical
Limber et al. [21]	Serious	Low	Low	Moderate	Serious	Moderate	Serious
Benitez-Sillero et al. [31]	Serious	Low	Moderate	Low	Serious	Low	Serious
Thompkins et al. [36]	Serious	Low	Serious	Moderate	Serious	Moderate	Serious
Sharma et al. [25]	Serious	Low	Moderate	Low	Serious	Moderate	Serious
Massey et al. [37]	Serious	Low	Serious	Moderate	Serious	Moderate	Serious
Kongsuwan et al. [38]	Critical	Low	Low	Low	Critical	Moderate	Critical
Suh (2023) [39]	Serious	Low	Moderate	Low	Serious	Moderate	Serious

**Table 7:** Effects of violence prevention programs.

Author	Year	Intervention	Exposure to Intervention	Measuring tools	Outcome Variable	Key Observations
Ando [33]	2007	Going Places Program (adapted)	4 lessons; 50 min per week for 4 weeks	Aggressive behavior items based on previous studies	Aggressive behavior	Significant reduction in aggressive behavior over time for both groups
						No observed differences between groups
Wolfe [22]	2009	Fourth R: Skills for young relationships	21 lessons of 75 min	National Longitudinal Survey of Children and Youth Inventory	Physical dating violence	Significant reduction in physical dating violence occurrence for the intervention group compared to the control group
						Greater intervention effect for boys
					Physical peer violence	
Castillo-Gualda [26]	2017	INTEMO	3 years; 12 lessons of 1 h each year	Aggression Questionnaire	Aggression	Lower levels of negative affect, feelings of anger and hostility, and physical and verbal violence in the intervention group compared to the control group
				Positive and Negative Affect Schedule	Negative affect	Negative affectivity, anger, and hostility mediated the effect of the intervention on aggression
Niolon [23]	2019	Dating Matters	4 years; 6–10 classroom sessions per year	Conflict in Adolescent Dating Relationships Inventory	Teen dating violence perpetration	Lower levels of teen dating violence perpetration and victimization and negative conflict resolution strategies in the intervention group compared to the control group across time points
				Conflict Resolution Styles Inventory (compliance; conflict engagement and withdrawal)	Teen dating violence victimization	
					Negative conflict resolution strategies	
Duncan [34]	2017	Positive Action Program	6 years	Social-Emotional and Character Development Scale	Social-emotional and character development	Overall decline in SECD and increase in misconduct over time across participants
				Aggression Scale	Misconduct	Significantly higher SECD and lower misconduct scores in the intervention group compared to the control group at the end point
				Frequency of Delinquent Behavior Scale		
Van Ryzin & Roseth [28]	2018	Cooperative learning		University of Illinois Bullying Scale	Bullying perpetration	No significant main effect on bullying
				Relatedness Scale	Bullying victimization	Significantly lower levels of bullying, victimization, and perceived stress on marginalized students in the intervention group
Valente [27]	2020	#Tamojunto	12 lessons of 50 min	EU-DAP item scale	School violence	Increase in school violence post-intervention mediated by a reduction in decision-making skills
Taylor [32]	2015	Shifting Boundaries	6 sessions over a period of 6–10 weeks	26-item scale	Sexual harassment perpetration	School-wide intervention showcased significant reduction in SH perpetration and victimization frequency and sexual dating violence victimization
					Sexual harassment victimization	No significant differences in the effects between gender and prior history groups
					Sexual dating violence perpetration	
					Sexual dating violence victimization	
Frank [35]	2014	Transformative Life Skills	3–4 lessons of 30 min per week during the first semester of the school year	Transgression-Related Interpersonal Motivations Scale	Revenge motivation	Significant post-intervention reduction in hostility and revenge motivation
				Brief Symptoms Inventory-18	Hostility	
						
Limber [21]	2018	Olweus Bullying Prevention Program		Olweus Bullying Questionnaire	Being bullied	Significant reduction in bullying perpetration and victimization across all grades
					Bullying others	Similar effects across genders and ethnicity
					Reactions to bullying	Contrasting results regarding the program’s strength: the being bullied results suggest greater effectiveness at lower grades, while the bullying others results suggest stronger effects at higher grades
Sulivan [29]	2016	Olweus Bullying Prevention Program; Second Step		Problem Behavior Frequency Scale	Overt aggression	No overall effect of intervention
				Children’s Anger Management Scale	Relational aggression	Mediating effect of gender: significant reduction in bullying by boys
					Bullying	
Niolon [24]	2024	Dating Matters		Conflict in Adolescent Dating Relationships Inventory	Teen dating violence perpetration	Lasting effects leading to reduction in teen dating violence and negative conflict resolution strategies
				Conflict Resolution Style Inventory (compliance; conflict engagement and withdrawal)	Teen dating violence victimization	
					Negative conflict resolution strategies	
Benítez-Sillero [31]	2020	PREBULLPE	6 lessons of 1 h	European Bullying Intervention Project Questionnaire	Bullying aggression	Decrease in cyberbullying victimization; no clear effect in cyberbullying aggression
				European Cyberbullying Intervention Project Questionnaire	Bullying victimization	No difference in intervention effects between genders
					Cyberbullying aggression	
					Cyberbullying victimization	
Thompkins [36]	2014	Violence Prevention Project	12 lessons of 45 min	Interpersonal Problem Solving Analysis	Verbal aggression	No effect on the likelihood of adopting prosocial conflict resolution strategies
					Physical aggression	Reduction in aggressive responses in the intervention group compared to the control group
					Antisocial behavior	
					Immature avoidance	
Sharma [25]	2020	Setu	4 lessons of 2 h	Illinois Bullying Scale	Victimization	Significant reduction in aggression and victimization levels
					Nonphysical aggression	The effect was greater at the 6-month follow-up rather than immediately after the intervention
					Physical aggression	Mediation effect of gender and age: greater effect for older students (13–14) and girls
Karmaliani [30]	2020	Right to Play	60 sessions of 35 min per year over 2 years	Peer-Victimization Scale	Peer victimization	Significant effect of intervention, resulting in a reduction in peer violence victimization and perpetration
				Peer-Perpetration Scale	Peer perpetration	
Massey [37]	2008	On-Campus Intervention Program			Frequency of disciplinary referrals	No intervention effect on the frequency of violent incidents
					Frequency of violence referrals	Significant reduction in dropout rates in the intervention group compared to the control group
					Dropout rate	
Konguswan [38]	2012	Orem’s Self-Care Deficit Theory of Nursing (adapted)	12 sessions of 1 h over 12 weeks	Aggressive Behaviors Scale (modified)	Attitudes toward violence	Significant decrease in adolescents’ aggressive attitudes post-implementation
				Observational Aggressive Behaviors Scale	Aggressive behavior	Increase in violence management skills
				Attitude Toward Violence Scale (modified)		
				Violence Management Skills Test		
Suh [39]	2023	Group Drumming	4 sessions of 50 min over 4 weeks	Aggression Questionnaire	Physical aggression	Significant intervention effect on anger subscale; no observed effect on aggression and hostility
					Verbal aggression	
					Anger	
					Hostility	

### 3.2. Meta-Analysis Results

**Figure 2:**
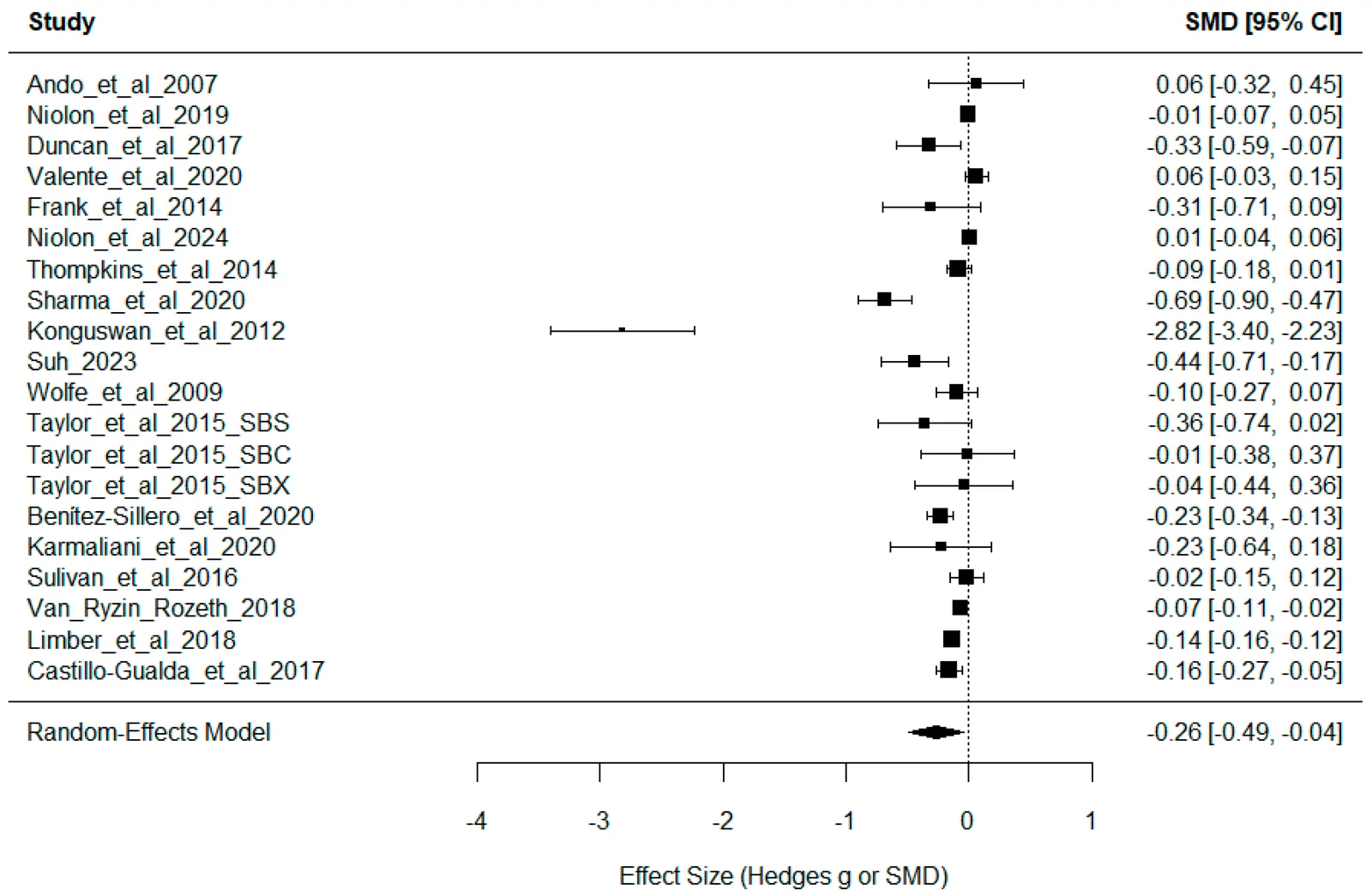
Aggregated model for violent behaviors k = 56 across 18 studies [21,22,23,24,25,26,27,28,29,30,31,32,33,34,35,36,38,39].

To account for the variance in study design and reported outcomes, a three-level random-effects meta-analytic model was used to assess the overall effect size of life skills interventions. The model was fitted to k = 56 effect sizes, drawn from 18 out of the 19 studies (Massey et al. did not provide sufficient information for effect sizes to be safely extracted). The model accounts for dependencies within studies by splitting variance into between-study heterogeneity (level 3) and within-study heterogeneity (level 2). The pooled effect size was statistically significant (*g* = −0.2652, SE = 0.1136, 95% CI [−0.4879, −0.0425], *z* = −2.3336, *p* = 0.0196), indicating a small to medium reduction in violent behaviors (Figure 2). Robust Variance Estimation using the CR2 sandwich estimator was applied on top of the three-level model. No substantial differences were noted (*g* = −0.265, SE = 0.113, t(18.9) = −2.34, *p* = 0.0301, 95% CI [−0.502, −0.0284]), reinforcing the pooled effect estimate.

High heterogeneity was observed in effect sizes across the samples (*Q*(55) = 263.58, *p* < 0.001). The overwhelming majority (98.02%) resided in the between-study (level 3) level (σ^2^3 = 0.2386), with minimal within-study (level 2) variance (σ^2^2 = 0.0026; 1.08% of total variance). Consequentially an aggregated two-level model produced similar results (k = 18 studies, g = −0.2641, SE = 0.1143, *p* = 0.0209, 95% CI [−0.4882, −0.0401], I^2^ = 99.23% and τ^2^ = 0.2431), indicating that the three-level and aggregation approaches produced convergent conclusions.

Three potential moderators of the pooled effect were individually examined: (1) outcome type (bullying perpetration/victimization, dating violence perpetration/victimization, hostility, misconduct, and nonphysical aggression and physical aggression); (2) study design (randomized controlled trial vs. quasi-experimental); and (3) risk-of-bias tier. Omnibus tests of each moderator were conducted using robust Wald tests (CR2, clustered by study) alongside the model-based QM test. For risk-of-bias moderation, tiers were expressed as “Low/Some concerns” (Low, Moderate, or Some Concerns) and “High/Critical” (Serious, High, or Critical) to normalize the different rating scales of RoB2 and ROBINS-I.

The omnibus test of outcome type as a moderator was non-significant (*p* = 0.5403), and the corresponding robust Wald test returned a degenerate result (negative denominator degrees of freedom), reflecting insufficient independent clusters per outcome category (several outcome levels were represented by only 1–2 studies) rather than a stable null finding. Study design and risk-of-bias tier showed similar point estimates in the expected direction (quasi-experimental and higher-risk-of-bias studies associated with larger apparent effects), but neither was statistically robust (F(1, 17) = 2.48; *p* = 0.134 and F(1, 17.1) = 2.87; *p* = 0.108, respectively).

The leave-one-out analysis identified Konguswan et al. [38] as a highly influential study: its exclusion reduced the model standard error from a typical range of 0.119–0.122 (observed for all other studies) to 0.0419 and produced the largest shift in the pooled point estimate of any single-study exclusion (from −0.2652 to −0.144). The study reported a disproportionately large effect size (g = −2.82, SE = 0.298; range of all other studies −0.1 to −0.4). The study was rated as “Critical” by ROBINS-I, due to critical ratings on the confounding and outcome measurement domains (Table 6).

Re-fitting the aggregated model without Konguswan et al. (k = 17 studies) produced a pooled effect size of g = −0.1422 (SE = 0.0421, 95% CI [−0.2246, −0.598], *p* = 0.0007), compared to g = −0.2641 (95% CI [−0.4882, −0.0401]) in the full sample. The direction and statistical significance of the pooled effect were preserved, but the magnitude was reduced by roughly 46%, and precision improved substantially (SE reduced by 63%). Between-study heterogeneity also declined (τ^2^ from 0.2431 to 0.0231; I^2^ from 99.23% to 92.82%), though it remained high by conventional benchmarks.

PIs of 95% were estimated using both the aggregated (two-level) and the sensitivity model, to characterize the range of the true effect in a future comparable study. The sensitivity model produced a significantly narrower PI, but in both models the computed intervals crossed 0 (95% PI [−0.4512, 0.1668] and 95% PI [−1.2560, 0.7278] for the sensitivity and aggregated models, respectively). Thus, the expected outcome of a new study could range from a moderate to low decrease in violent behaviors to a low increase.

The trim-and-fill method imputed zero missing studies in both the full sample and the sensitivity sample, excluding Konguswan et al., providing no evidence of asymmetry attributable to missing studies under this method. Egger’s regression test, applied to the aggregated study-level data, was non-significant in both samples (full: t(18) = −1.0197, *p* = 0.3214; sensitivity: t(17) = −0.2973, *p* = 0.7698). Begg’s rank correlation test produced similar results in the full sample (Kendall’s τ = −0.2526, *p* = 0.1284) and in the sensitivity one (τ = −0.1696, *p* = 0.3320).

## 4. Discussion

The current review examined the effectiveness of life skills-based violence prevention programs in secondary education. For this purpose, 19 studies were identified, five focused on bullying prevention, four on dating violence prevention, and the remaining 10 on general aggression/violence prevention. Most studies report positive results, indicating an overall effectiveness of programs implemented during middle and high school. Similar results are reported in another review [40].

General aggression/violence-focused studies reported the most mixed results, with half of them reporting no clear intervention effect [27,33,36,37,39]. In contrast, all bullying and dating violence prevention programs reported positive main or moderated effects. This could reflect the heterogeneity in how “general violence” is conceptualized and measured or suggest greater strength for programs targeting specific domains of violence.

Across the reviewed interventions and programs, the life skills components most consistently linked to positive outcomes were emotional regulation, conflict resolution, problem solving, communication, and empathy. These findings align with theoretical frameworks that identify deficits in self-regulation and interpersonal competence as risk factors for violence perpetration [12]. Notably, three studies demonstrated explicit mediation pathways: Castillo-Gualda et al. [26] identified emotional regulation as a mediator of reduced aggression; Valente et al. [27] found that reduction in decision-making skills mediated adverse program effects; and Van Ryzin & Roseth [28] showed that cooperative peer relationships mediated reductions in bullying victimization. These findings align with previous research that negatively associated social skills and executive functioning (such as cooperation, emotional regulation, and problem solving) with bullying and aggressive behaviors [41,42,43], and showcase the importance of targeting specific, theoretically grounded mechanisms.

Furthermore, long-term persistence of intervention effects remains largely unverified. Only three eligible studies reported post-intervention follow-up outcomes. Because follow-up time points were rarely measured and inconsistently reported, no pooled meta-analytic estimate could be computed for long-term outcomes. Consequently, no firm conclusions can be drawn regarding whether the immediate post-intervention reductions in violence are sustained over time, representing a key gap for future longitudinal research.

Gender moderation showed some significance across studies. The Fourth R program [22] and the OBPP/Second Step [29] showed greater effect on reducing dating violence and bullying, respectively, for boys. On the other hand, the Right to Play program [30] and the Setu program [25] resulted in a greater reduction in violent behavior in girls. Inconclusive results have been previously described [44,45], which are attributed to the inconsistency of reporting the sociodemographic characteristics across studies.

Age was also a moderating factor in the effectiveness of Setu, with greater effects observed for older students. Interestingly, Limber et al. [21] report differential age moderation across grades; bullying victimization rates improved more in lower grades, while perpetration rates improved more in higher grades. These findings suggest that developmental stage and gender should be considered factors when constructing a program curriculum, though further research is required to identify the reasoning behind these observations.

A consistent, statistically significant pooled effect observed in both the three-level mixed effects and the aggregated two-level models supports the effectiveness of the interventions. However, the extreme heterogeneity, which is almost entirely between studies (I^2^ ≈ 99%), accompanying the pooled estimate indicates that a single effect size poorly describes the existing literature and should be interpreted alongside the extreme variability, rather than instead of it. The computed prediction intervals, showing the possible range of effects for a future study, reinforce this statement. While the overall pooled effect can be expected to remain positive (both 95% CI below 0), successful implementation of a new comparable intervention is not guaranteed, as both 95% PIs for the aggregated and sensitivity models cross 0.

Of the four moderators examined (outcome, study design, risk of bias, and sample size), none could explain the heterogeneity observed in the sample. However, this result should be interpreted cautiously, rather than considered conclusive evidence of non-moderation, as moderator tests are quite unstable under low cluster numbers.

A disproportionate influence in leave-one-out analysis, an order of magnitude higher effect size than the rest, an independent ROBINS-I “Critical” score, and the near collapse of the between-study variance component in Konguswan et al. [38] identify the study as a key driver of both the magnitude and the heterogeneity of the pooled estimate. The exclusion of the study resulted in a still significant but reduced by 46% pooled effect and an 63% drop in between-study variance. Due to all possible moderators being confounded in this single study (quasi-experimental design, high risk of bias), it is not possible to identify if any of these factors are responsible for the observed result. Given the drop in variance, the sensitivity estimate (g = −0.1422, 95% CI [−0.2246, −0.0598]) is likely the more stable and generalizable summary of the broader literature, though it should be noted that heterogeneity is still high (I^2^ ≈ 93%).

Rather than an artifact of incomplete moderator coverage or insufficient statistical power, the near-total between-study heterogeneity (I^2^ ≈ 99%) could plausibly reflect genuine theoretical and methodological fragmentation within this specific literature. The included interventions span markedly different theoretical traditions, delivered at doses ranging from approximately 2–3 h of total contact to several years of programming, and were measured with a wide range of self-report, observational, and administrative record instruments, even within a single outcome category. Given this, a single pooled effect size across the full set of studies should be understood as a broad, approximate summary of the conceptually heterogeneous literature rather than a precise estimate of one underlying treatment effect. We would caution readers against over-interpreting the point estimate’s apparent precision (reflected in a narrow confidence interval) as evidence that the underlying effect is similarly homogeneous or well-estimated.

This reframing also shifts where meaningful progress is likely to come from. Because study-level moderator analyses are inherently limited by the small number of independent clusters available (k = 19) and by the fact that several plausible sources of variation are not routinely reported in the primary literature (e.g., stratified effects by gender, precise dose/duration, follow-up interval, and comparator type), further secondary synthesis of the existing evidence base is unlikely to resolve the heterogeneity on its own. Progress is more likely to depend on changes to how future primary research in this specific field is designed and reported.

This review has several limitations. As expected, restriction to secondary education interventions returned a limited number of studies (~90 studies excluded due to not meeting the age requirements). Furthermore, language restriction in the selection criteria could have further limited the available pool of studies. This results in large heterogeneity between study characteristics and outcomes, as shown in the results section, with prevention programs represented only once, making comparisons ambiguous and more exploratory meta-analysis not feasible. The limited number of studies could also lead to circumstantial evidence, rather than conclusive results in areas such as gender/age moderation and sustained intervention effects. Moreover, the limited number of studies leads to unstable moderator analysis results, due to low cluster numbers (e.g., negative denominator degrees of freedom in the outcome measure Wald test). As such, these results should be interpreted as indicators, rather than proof of evidence of null moderation. Uneven geographic representation, with most studies being conducted in the US, limits the generalizability of the results to different cultural contexts. The overall low quality of the studies, though anticipated, should also be considered. The self-reporting nature of the assessments, especially when students are not blind to the intervention, could easily lead to inflated effect sizes. Also, failure to account for confounders in pre–post quasi-experimental designs with nonequivalent control groups could produce false positive results. A critical methodological issue identified in this review is the widespread lack of prospective study pre-registration. Only 3 of the 19 eligible studies provided evidence of pre-registration. The absence of pre-registration across the vast majority of included studies introduces a notable risk of reporting bias and selective outcome reporting. In un-registered studies, researchers retain post hoc flexibility in defining primary versus secondary outcomes, choosing analytical models, or deciding which specific subscales and time points to report. This operational freedom significantly heightens the risk of p-hacking and inflation of positive effect sizes.

## 5. Conclusions

The findings of this review provide evidence for the integration of life skills-based violence prevention programs into secondary school curricula, particularly programs that focus on emotional regulation, conflict resolution, and empathy. Moreover, though not present across the whole study pool, moderation effects for age and gender were reported. However, due to several limitations discussed above (extreme between-study heterogeneity, overall poor quality, and the small number of included studies) these results should be interpreted cautiously, and the practical implications of such findings remain inconclusive. Future research should prioritize randomized controlled designs with longer follow-up periods, objective outcome measures, and pre-registered analysis plans to minimize bias. Greater geographic diversity in study samples is needed to assess the cross-cultural generalizability of program effects. Further research into the relative contribution of individual life skills components would enhance the field’s understanding of the active mechanisms of change, leading to the construction of more robust curricula. Finally, cost-effectiveness analyses would provide valuable guidance for policy decisions around program adoption.

## Figures and Tables

**Table 1:** PICOS algorithm for the systematic review.

Participants	Intervention	Comparison	Outcomes	Study Design
Adolescents 12–18 years old or/and students in secondary education	School-based violence prevention programs through life skills education	Before and after the intervention or between intervention groups and control groups	Effectiveness in reducing violent and aggressive behaviors	Interventional

**Table 2:** Inclusion criteria for the systematic review.

Inclusion Criteria
Studies assessed the effectiveness of school-based violence prevention programs through life skills training as a key intervention component (interventional studies). Only interventional studies were considered eligible. Eligible studies examined school-based intervention programs that aimed to develop adolescents’ life skills (e.g., emotional regulation, communication, empathy, problem solving, and conflict resolution) with the explicit goal of reducing school violence or aggression. Studies provided data related to school violence, including aggressive behavior, bullying, victimization, or dating/relationship violence. Participants were adolescents or secondary school students, aged 12 to 18 years, or studies reporting a mean age of participants within this range. Interventions were carried out within the school, either during daily activities or as part of specific prevention initiatives undertaken by the school. Studies involving mixed groups of adolescents and adults were included only if they provided separate data for adolescent participants. Articles written in English or Greek. Studies published between January 2005 and May 2025.

**Table 3:** Exclusion criteria for the systematic review.

Exclusion Criteria
Case reports, books, encyclopedias, news, short communications, reviews, and observational studies. Review articles or theoretical/medical hypothesis papers. Studies that did not specify participant age groups. Studies involving participants younger than 12 or older than 18 years. Studies that used questionnaires that did not directly assess the effectiveness of the programs. Studies in which parents completed questionnaires for their children. Studies that focused primarily on high-risk behaviors outside of school, such as alcohol or drug use, HIV prevention, or sexual health issues, unless they had measurable outcomes related to violence. Studies that focused solely on cyberbullying without assessing in-school violence or aggression. Studies not published in English or Greek.

## Data Availability

The review protocol was registered with Inplasy (registration number: INPLASY202650168). The DOI number is 10.37766/inplasy2026.5.0168. INPLASY.COM.

## Supplementary Materials

The following supporting information can be downloaded at: https://www.mdpi.com/article/10.3390/children13091128/s1.

## References

[1] World Health Organization (2018). Adolescent Health Epidemiology.

[2] Ostrov J.M., Blakely-McClure S.J., Perry K.J., Kamper-DeMarco K.E., Coyne S.M., Ostrov J.M. (2018). Definitions: The form and function of relational aggression. The Development of Relational Aggression.

[3] Allen J.J., Anderson C.A., Sturmey P. (2017). Aggression and violence: Definitions and distinctions. The Wiley Handbook of Violence and Aggression.

[4] Ortega-Ruiz R., Del Rey R., Casas J.A. (2016). Evaluar el bullying y el cyberbullying: Validación española del EBIP-Q y del ECIP-Q. Psicol. Educ..

[5] Lan M., Law N., Pan Q., Gaffney H., Farrington D.P., Espelage D.L., Ttofi M.M., Fekkes M., van de Sande M.C.E., Gravesteijn J.C. (2022). Evaluation of a whole-school change intervention: Findings from a two-year cluster-randomized trial of the restorative practices intervention. Comput. Educ..

[6] Yokoyama M., Suzuki M., Takai Y., Igarashi A., Noguchi-Watanabe M., Yamamoto-Mitani N. (2016). Workplace bullying among nurses and their related factors in Japan: A cross-sectional survey. J. Clin. Nurs..

[7] Turner M.G., Exum M.L., Brame R., Holt T.J. (2013). Bullying victimization and adolescent mental health: General and typological effects across sex. J. Crim. Justice.

[8] Farrell A.D., Sullivan T.N., Esposito L.E., Meyer A.L., Valois R.F. (2005). A latent growth curve analysis of the structure of aggression, drug use, and delinquent behaviors and their interrelations over time in urban and rural adolescents. J. Res. Adolesc..

[9] Hazler R.J. (1996). Breaking the Cycle of Violence: Interventions for Bullying and Victimization.

[10] World Health Organization (1997). Life Skills Education for Children and Adolescents in Schools.

[11] World Health Organization (2003). Skills for Health: Skills-Based Health Education Including Life Skills: An Important Component of a Child-Friendly/Health-Promoting School.

[12] Dahlberg L.L., Simon T.R., Lutzker J.R. (2006). Predicting and preventing youth violence: Developmental pathways and risk. Preventing Violence: Research and Evidence-Based Intervention Strategies.

[13] World Health Organization (2009). Preventing Violence by Developing Life Skills in Children and Adolescents.

[14] Page M.J., McKenzie J.E., Bossuyt P.M., Boutron I., Hoffmann T.C., Mulrow C.D., Shamseer L., Tetzlaff J.M., Akl E.A., Brennan S.E. (2021). The PRISMA 2020 statement: An updated guideline for reporting systematic reviews. BMJ.

[15] Sterne J.A.C., Savović J., Page M.J., Elbers R.G., Blencowe N.S., Boutron I., Cates C.J., Cheng H.-Y., Corbett M.S., Eldridge S.M. (2019). RoB 2: A revised tool for assessing risk of bias in randomised trials. BMJ.

[16] Chinn S. (2000). A simple method for converting an odds ratio to effect size for use in meta-analysis. Stat. Med..

[17] Polanin J.R., Snilstveit B. (2016). Converting between effect sizes. Campbell Syst. Rev..

[18] Viechtbauer W. (2010). Conducting meta-analyses in R with the metafor package. J. Stat. Softw..

[19] Pustejovsky J.E. (2022). ClubSandwich: Cluster-Robust (Sandwich) Variance Estimators with Small-Sample Corrections [R Package]. https://CRAN.R-project.org/package=clubSandwich.

[20] Egger M., Smith G.D., Schneider M., Minder C. (1997). Bias in meta-analysis detected by a simple, graphical test. BMJ.

[21] Limber S.P., Olweus D., Wang W., Masiello M., Breivik K. (2018). Evaluation of the Olweus Bullying Prevention Program: A large scale study of U.S. students in grades 3–11. J. Sch. Psychol..

[22] Wolfe D.A., Crooks C., Jaffe P., Chiodo D., Hughes R., Ellis W., Stitt L., Donner A. (2009). A school-based program to prevent adolescent dating violence: A cluster randomized trial. Arch. Pediatr. Adolesc. Med..

[23] Niolon P.H., Vivolo-Kantor A.M., Tracy A.J., Latzman N.E., Little T.D., DeGue S., Lang K.M., Estefan L.F., Ghazarian S.R., McIntosh W.L.K. (2019). An RCT of Dating Matters: Effects on teen dating violence and relationship behaviors. Am. J. Prev. Med..

[24] Niolon P.H., Estefan L.F., DeGue S., Le V.D., Tracy A.J., Ray C., Bontempo D., Little T.D., Vivolo-Kantor A.M., Latzman N. (2024). High school follow-up of the Dating Matters® RCT: Effects on teen dating violence and relationship behaviors. Prev. Sci..

[25] Sharma D., Mehari K.R., Kishore J., Sharma N., Duggal M. (2020). Pilot evaluation of Setu, a school-based violence prevention program among Indian adolescents. J. Early Adolesc..

[26] Castillo-Gualda R., Cabello R., Herrero M., Rodríguez-Carvajal R., Fernández-Berrocal P. (2018). A three-year emotional intelligence intervention to reduce adolescent aggression: The mediating role of unpleasant affectivity. J. Res. Adolesc..

[27] Valente J.Y., Cogo-Moreira H., Sanchez Z.M. (2020). Decision-making skills as a mediator of the #Tamojunto school-based prevention program: Indirect effects for drug use and school violence of a cluster-randomized trial. Drug Alcohol Depend..

[28] Van Ryzin M.J., Roseth C.J. (2018). Cooperative learning in middle school: A means to improve peer relations and reduce victimization, bullying, and related outcomes. J. Educ. Psychol..

[29] Sullivan T., Sutherland K., Farrell A., Taylor K., Doyle S. (2017). Evaluation of violence prevention approaches among early adolescents: Moderating effects of disability status and gender. J. Child Fam. Stud..

[30] Karmaliani R., McFarlane J., Khuwaja H.M.A., Somani Y., Bhamani S.S., Ali T.S., Asad N., Chirwa E.D., Jewkes R. (2020). Right To Play’s intervention to reduce peer violence among children in public schools in Pakistan: A cluster-randomized controlled trial. Glob. Health Action.

[31] Benítez-Sillero J.D., Corredor-Corredor D., Córdoba-Alcaide F., Calmaestra J. (2021). Intervention programme to prevent bullying in adolescents in physical education classes (PREBULLPE): A quasi-experimental study. Phys. Educ. Sport Pedagog..

[32] Taylor B.G., Mumford E.A., Stein N.D. (2015). Effectiveness of “Shifting Boundaries” teen dating violence prevention program for subgroups of middle school students. J. Adolesc. Health.

[33] Ando M., Asakura T., Ando S., Simons-Morton B. (2007). A psychoeducational program to prevent aggressive behavior among Japanese early adolescents. Health Educ. Behav..

[34] Duncan R., Washburn I.J., Lewis K.M., Bavarian N., DuBois D.L., Acock A.C., Vuchinich S., Flay B.R. (2017). Can universal SEL programs benefit universally? Effects of the Positive Action Program on multiple trajectories of social-emotional and misconduct behaviors. Prev. Sci..

[35] Frank J.L., Bose B., Schrobenhauser-Clonan A. (2014). Effectiveness of a school-based yoga program on adolescent mental health, stress coping strategies, and attitudes toward violence: Findings from a high-risk sample. J. Appl. Sch. Psychol..

[36] Thompkins A.C., Chauveron L.M., Harel O., Perkins D.F. (2014). Optimizing violence prevention programs: An examination of program effectiveness among urban high school students. J. Sch. Health.

[37] Massey O.T., Boroughs M., Armstrong K.H. (2008). School violence interventions in the Safe Schools/Healthy Students initiative: Evaluation of two early intervention programs. J. Sch. Violence.

[38] Kongsuwan V., Suttharangsee W., Isaramalai S., Weiss S.J. (2012). The development and effectiveness of a violence prevention program for Thai high school adolescents. Pac. Rim Int. J. Nurs. Res..

[39] Suh E.S. (2023). The use of group drumming-based music therapy with male adolescents in a school violence prevention program in Korea: A pilot study. Psychol. Music.

[40] Muser J., Whitten T., Virgara J., Connell N., Tzoumakis S. (2025). Can universal school-based social and emotional learning programs reduce early antisocial behaviours? A systematic review and meta-analysis. Int. J. Behav. Dev..

[41] Eisenberg N., Carlo G., Murphy B., van Court P. (1995). Prosocial Development in Late Adolescence: A Longitudinal Study. Child Dev..

[42] Jenkins L.N., Demaray M.K., Fredrick S.S., Summers K.H. (2016). Associations Among Middle School Students’ Bullying Roles and Social Skills. J. Sch. Violence.

[43] Mahady Wilton M.M., Craig W.M., Pepler D.J. (2000). Emotional Regulation and Display in Classroom Victims of Bullying: Characteristic Expressions of Affect, Coping Styles and Relevant Contextual Factors. Soc. Dev..

[44] Clarke A., Sorgenfrei M., Mulcahy J., Davie P., Friedrich C., McBride T. (2021). Adolescent Mental Health: A Systematic Review on the Effectiveness of School-Based Interventions. Early Intervention Foundation. https://www.eif.org.uk/report/adolescent-mental-health-a-systematic-review-on-the-effectiveness-of-school-based-interventions.

[45] Durlak J.A., Mahoney J.L., Boyle A.E. (2022). What we know, and what we need to find out about universal, school-based social and emotional learning programs for children and adolescents: A review of meta-analyses and directions for future research. Psychol. Bull..

