# Supplementary material for: The Effectiveness of Life Skills-Based School Violence Prevention Programs in Secondary Education: A Systematic Review and Meta-Analysis
Source: Children (Basel). 2026 Aug 23;13(9):1128. doi: 10.3390/children13091128 (PMC13605921; doi:10.3390/children13091128)
Supplement: Supplementary file 1 [file children-13-01128-s001.zip › Supplementary S1.pdf]

Suppl. Table S1: Search Algorithms used for each database

Database	Algorithm
PubMed	(“Schools"[MeSH] OR school-based[tiab] OR school based[tiab]) AND ("Primary Prevention"[MeSH] OR prevention[tiab]) AND (program*[tiab] OR intervention*[tiab] OR practice*[tiab] OR module*[tiab] OR workshop*[tiab] OR strategy*[tiab] OR approach*[tiab]) AND ("Violence"[MeSH] OR "Aggression"[MeSH] OR violence[tiab] OR violent[tiab] OR aggression[tiab] OR stress[tiab]) AND ("Life Skills"[MeSH] OR "Social Skills"[MeSH] OR "Emotional Intelligence"[MeSH] OR life skills[tiab] OR life-skills interventions[tiab] OR empathy[tiab] OR self awareness[tiab] OR compassion[tiab] OR sympathy[tiab] OR critical thinking[tiab] OR creative thinking[tiab] OR decision making[tiab] OR problem solving[tiab] OR emotion management[tiab] OR emotional control[tiab] OR emotional intelligence[tiab] OR social emotional learning[tiab] OR relationship management[tiab] OR relationship building[tiab] OR stress management[tiab] OR stress regulation [tiab] OR resistant to social pressure*[tiab] OR communication skills[tiab] OR interpersonal skills[tiab] OR social competence[tiab] OR emotional competence[tiab] OR emotion regulation[tiab] OR self-regulation[tiab] OR coping skill*[tiab] OR resilience[tiab] OR prosocial behavior[tiab] OR behavior regulation[tiab] OR conflict resolution[tiab] OR anger management[tiab]) AND ("Adolescent"[MeSH] OR secondary education[tiab] OR adolescent*[tiab] OR teenager*[tiab] OR adolescence[tiab] OR teen*[tiab] OR puberty[tiab] OR student*[tiab] OR high school[tiab]) AND ("Program Evaluation"[MeSH] OR "Treatment Outcome"[MeSH] OR effectiveness[tiab] OR impact[tiab] OR outcome*[tiab])
Scopus	TITLE-ABS-KEY (("school-based" OR "school based") AND (prevention) AND (program* OR intervention* OR practice* OR module* OR workshop* OR strategy* OR approach*) AND (violence OR violent OR aggression OR stress) AND ("life skills" OR "life-skills interventions" OR empathy OR "self awareness" OR compassion OR sympathy OR "critical thinking" OR "creative thinking" OR "decision making" OR "problem solving" OR "emotion management" OR "emotional control" OR "emotional intelligence" OR "social emotional learning" OR "relationship management" OR "relationship building" OR "stress management" OR "stress regulation" OR "resistant to social pressure*" OR "communication skills" OR "interpersonal skills") AND ("secondary education" OR adolescent* OR teenager* OR adolescence OR teen* OR puberty OR student* OR "high school") AND (effectiveness OR impact OR outcome*))
CINAHL	(MH Schools+ OR XB (school-based OR school based)) AND (MH Prevention+ OR XB prevention) AND (XB (program* OR intervention* OR practice* OR module* OR workshop* OR strategy* OR approach*)) AND (MH Violence+ OR MH Aggression+ OR MH Stress+ OR XB (violence OR violent OR aggression OR stress)) AND (MH Life Skills+ OR MH Social Skills+ OR MH Emotional Intelligence+ OR XB (life skills OR life-skills interventions OR empathy OR self awareness OR compassion OR sympathy OR critical thinking OR creative thinking OR decision making OR problem solving OR emotion management OR emotional control OR emotional intelligence OR social emotional learning OR relationship management OR relationship building OR stress management OR stress regulation OR resistant to social pressure* OR communication skills OR interpersonal skills)) AND (MH Adolescence+ OR MH Students+ OR MH Secondary Education+ OR XB (secondary education OR adolescent* OR teenager* OR adolescence OR teen* OR puberty OR student* OR high school)) AND (MH Program Evaluation+ OR MH Treatment Outcomes+ OR XB (effectiveness OR impact OR outcome*))
ERIC	(SUBJECT(School Based Programs) OR NOFT(school-based OR school based)) AND (SUBJECT(Prevention) OR NOFT(prevention)) AND (NOFT(program* OR intervention* OR practice* OR module* OR workshop* OR strategy* OR approach*)) AND (SUBJECT(School Violence OR Violence OR Aggression) OR NOFT(violence OR violent OR aggression OR stress)) AND (SUBJECT(Life Skills OR Social Emotional Learning OR Empathy OR Social Skills) OR NOFT(life skills OR life-skills interventions OR empathy OR self awareness OR compassion OR sympathy OR critical thinking OR creative thinking OR decision making OR problem solving OR emotion management OR emotional control OR emotional intelligence OR social emotional learning OR relationship

	management OR relationship building OR stress management OR stress regulation OR resistant to social pressure* OR communication skills OR interpersonal skills OR social competence OR emotional competence OR emotion regulation OR self-regulation OR coping skill* OR resilience OR prosocial behavior OR behavior regulation OR conflict resolution OR anger management)) AND (SUBJECT(Secondary Education OR Adolescents OR Secondary School Students) OR NOFT(secondary education OR adolescent* OR teenager* OR adolescence OR teen* OR puberty OR student* OR high school)) AND (SUBJECT(Program Effectiveness OR Outcomes of Education) OR NOFT(effectiveness OR impact OR outcome*))
PsycInfo	(DE Schools OR XB (school-based OR school based)) AND (DE Prevention OR XB prevention) AND (XB (program* OR intervention* OR practice* OR module* OR workshop* OR strategy* OR approach*)) AND (DE Violence OR DE Aggression OR DE Stress OR XB (violence OR violent OR aggression OR stress)) AND (DE Life Skills OR DE Social Skills OR DE Emotional Intelligence OR DE Empathy OR DE Emotional Regulation OR XB (life skills OR life-skills interventions OR empathy OR self awareness OR compassion OR sympathy OR critical thinking OR creative thinking OR decision making OR problem solving OR emotion management OR emotional control OR emotional intelligence OR social emotional learning OR relationship management OR relationship building OR stress management OR stress regulation OR resistant to social pressure* OR communication skills OR interpersonal skills OR social competence OR emotional competence OR emotion regulation OR self-regulation OR coping skill* OR resilience OR prosocial behavior OR behavior regulation OR conflict resolution OR anger management)) AND (DE Adolescence OR DE Students OR DE Secondary Education OR XB (secondary education OR adolescent* OR teenager* OR adolescence OR teen* OR puberty OR student* OR high school)) AND (DE Program Evaluation OR DE Treatment Outcomes OR XB (effectiveness OR impact OR outcome*))

Suppl. Table S2: Quality assessment of randomized controlled trials using Cochrane RoB2.

Study	D1: Randomization	D2: Deviations	D3: Missing Data	D4: Measurement	D5: Reporting	Overall
Wolfe (2009)	Some concerns	Low	Some concerns	Some concerns	Low	**Some concerns**
Niolon (2019)	Some concerns	Low	Some concerns	Some concerns	Low	**Some concerns**
Duncan (2017)	Some concerns	Low	Some concerns	High	Some concerns	**High**
Van Ryzin & Roseth (2018)	Some concerns	Low	Low	Some concerns	Low	**Some concerns**
Valente (2020)	Low	Low	Some concerns	Some concerns	Some concerns	**Some concerns**
Taylor (2015)	Some concerns	Some concerns	Low	Some concerns	Low	**Some concerns**
Sullivan (2017)	High	Some concerns	Some concerns	Some concerns	Some concerns	**High**
Niolon (2024)	Some concerns	Low	Some concerns	Some concerns	Low	**Some concerns**
Karmaliani (2020)	Low	Low	Low	Some concerns	Low	**Low**

Suppl. Table S3: Quality assessment of quasi-experimental studies using Cochrane ROBINS-I V2.

Study	D1: Confounding	D3: Classification	D2: Selection	D5: Missing Data	D6: Measurement	D7: Reporting	Overall
Ando (2007)	Serious	Low	Moderate	Moderate	Serious	Moderate	Serious
Castillo-Gualda (2018)	Serious	Low	Moderate	Moderate	Serious	Moderate	Serious
Frank (2014)	Critical	Low	Serious	Moderate	Serious	Moderate	Critical
Limber (2018)	Serious	Low	Low	Moderate	Serious	Moderate	Serious
Benitez-Sillero (2021)	Serious	Low	Moderate	Low	Serious	Low	Serious
Thompkins (2014)	Serious	Low	Serious	Moderate	Serious	Moderate	Serious
Sharma (2020)	Serious	Low	Moderate	Low	Serious	Moderate	Serious
Massey (2007)	Serious	Low	Serious	Moderate	Serious	Moderate	Serious
Kongsuwan (2012)	Critical	Low	Low	Low	Critical	Moderate	Critical
Suh (2023)	Serious	Low	Moderate	Low	Serious	Moderate	Serious
